# Mirror Hand Disorder’s Surgical Management with Metacarpal Wedging

**DOI:** 10.3390/life14050588

**Published:** 2024-05-04

**Authors:** Zsombor Márton, Tamás Kassai, Aba Lőrincz, Gergő Józsa

**Affiliations:** 1 Department of Anatomy, Medical School, University of Pécs, 12 Szigeti Street, H7624 Pécs, Hungary; martonzsomi@gmail.com; 2 Department of Pediatric Traumatology, Péterfy Hospital, Manninger Jenő National Trauma Center, 17 Fiúmei Street, H1081 Budapest, Hungary; kassai.tamas@obsi.hu; 3 Department of Thermophysiology, Institute for Translational Medicine, Medical School, University of Pécs, 12 Szigeti Street, H7624 Pécs, Hungary; aba.lorincz@gmail.com; 4 Division of Surgery, Traumatology, Urology, and Otorhinolaryngology, Department of Pediatrics, Clinical Complex, University of Pécs, 7 József Attila Street, H7623 Pécs, Hungary

**Keywords:** paediatric, mirror hand disorder, ulnar dimelia, hand surgery, reconstructive surgery, hand malformation

## Abstract

Ulnar dimelia, also known as “mirror hand disorder”, is a rare developmental disorder affecting the upper limb. Primarily, it involves the duplication of fingers, carpal bones, metacarpals, or ulna along the sagittal axis, and is often accompanied by the absence of the radius or thumb. The anomaly presents challenges in both bone and soft tissue development, impacting limb functionality and affecting a child’s quality of life. We present the case of a one-year-old girl with unilateral ulnar dimelia. Surgical intervention was considered to address functional and aesthetic concerns. The surgery involved creating an opposable thumb from preaxial fingers through a carefully tailored approach. Post surgical therapy included physiotherapy and psychotherapy to ensure both physical functionality and psychological adjustment. The surgical procedure successfully provided an adequate grip pattern, and the patient demonstrated age-appropriate use of the modified hand at the six-month follow-up. Comparison with similar cases highlights the diversity in ulnar dimelia presentations and the need for customised surgical solutions. The timing of surgery is typically recommended between one and two years, considering both anatomical readiness and the advantages of cerebral plasticity in young patients.

## 1. Introduction

Ulnar dimelia, also referred to as “mirror hand disorder”, is one of the most unique developmental disorders affecting the upper limb. It is characterised by duplication of the fingers, carpal bones, metacarpals, and ulna along the sagittal axis, and the absence of the radius or thumb. Thus, the anomaly includes a symmetrical polydactyly and a complete absence of the thenar muscles [1]. In a typical ulnar dimelia case, the forearm and the hand show some degree of a mirrored image along their sagittal axis. However, there was a published case in which the deformity was reported without an axis of symmetry [2]. In this anomaly, the malformation of the bones is usually followed by the development of the surrounding soft tissues. Numerous cases have reported duplication of the ulnar artery or nerve, the appearance of an abnormal palmar arch, the absence of the radial artery, and shortening of the radial nerve [3]. In most of the reported cases, the triphalangeal radial (preaxial) fingers are more pronated and in a different plane than the ulnar (postaxial) fingers, which is called ulnar deviation. This is caused by weakness of the forearm extensors [1]. As a result of the abnormal anatomy, the ranges of flexion of the elbow joint and dorsiflexion or pronation of the wrist are significantly reduced. Due to the lack of thenar muscles, the opposition is not possible. These deformities significantly affect the functionality of the limb and therefore the child’s quality of life [4].

In 1852, the first documented case was reported, and the preparation of this limb is still preserved in the Warren Anatomical Museum at Harvard University [5]. Due to the rarity of the disorder, incidence data are not available. Approximately only 70 cases have been recorded in the literature [6]. To our knowledge, no Hungarian case has been described before.

Ulnar dimelia’s prevalence is the same in both genders [7]. It most commonly affects the left upper limb unilaterally without other developmental abnormalities. However, it may rarely also occur as part of a genetic condition, such as Laurin–Sandrow Syndrome. It is an autosomal dominant inherited disorder characterised by a bilateral ulnar and tibial duplication, with an absent radius and fibula [8,9,10,11].

Swanson summarised the classification of congenital hand deformities in 1976 [12]. This categorisation is still used today by both the American Society for Hand Surgery (ASSH) and the International Federation of Societies for Hand Surgery (IFSSH), which define ulnar dimelia as type III duplication [13] (Table 1).

Al-Qattan classified the abnormality into a spectrum, where he distinguished five main types based on the deformity of the bony structure of the forearm [1] (Table 2).

The main goal of the therapy is to achieve the appropriate functionality and aesthetics. This involves reducing the number of preaxial fingers and reconstructing an opposable thumb, which is known as pollicisation [14]. The exact timing of the operation is still a matter of scientific debate because with less than 101 cases worldwide, it is unlikely that the exact timing can be defined. However, it is important to perform the surgery when the patient is as young as possible to take advantage of the patient’s cerebral plasticity to achieve the best future functionality. However, the relatively small neurovascular structures associated with young age are a limiting factor. For this reason, it is recommended to perform the operation above the age of 12 months [15] but before the age of two years [16].

## 2. Materials and Methods

### 2.1. Case Description

The case of a 1-year-old girl who was admitted to our clinic with left ulnar dimelia is presented by the authors. She was delivered by caesarean section without complications. No upper limb malformations were noted during the prepartum ultrasound examination. The abnormality was detected after birth. In the first few days, she was diagnosed with a right-sided inguinal hernia, which was managed by surgery at our hospital. The literature does not indicate any connection between the two pathologies, so it is likely that the hernia is not associated with the upper limb deformity. No other congenital anomaly was confirmed during the examination of the patient.

**Figure 1:**
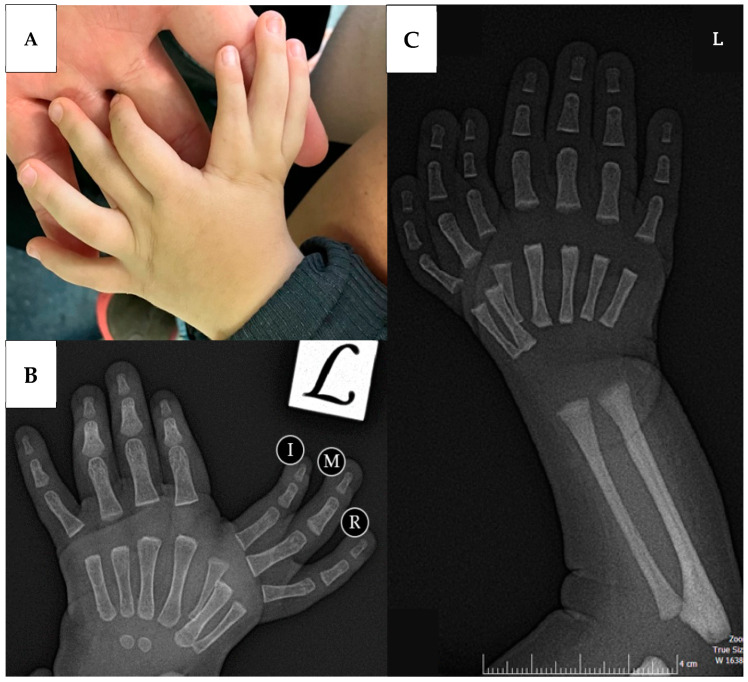
An image of the child’s left hand during the physical examination, where four postaxial and three ulnarly deviated preaxial triphalangeal fingers are visible (**A**). The pre-operative X-ray in the antero-posterior (AP) position of the patient’s left hand demonstrating a hypoplastic metacarpus of I and well-developed M and R fingers (**B**). The pre-operative (AP) radiograph of the child’s left forearm showing a properly formed ulna and radius (**C**).

Physical examinations revealed the following findings: The right upper limb was well developed for her age, with no abnormalities. On the left hand, seven triphalangeal fingers were observed, four of which were postaxial and three were preaxial (Figure 1A). Development of the postaxial fingers was considered adequate and their range of motion was sufficient, in contrast to the preaxial fingers, which were deviated in the ulnar direction, and whose range of motion was significantly reduced. Opposition was not possible due to the absence of the thumb and the first CMC joint. When the wrist and elbow joints were examined, the ranges of motion were found to be adequate for the elbow’s flexion–extension, pronation–supination, and palmar–dorsal flexion. No significant differences were observed between the movements of these joints of the two upper limbs.

After the physical examination, X-rays were taken of the patient’s hand in anterior–posterior and lateral view. A radiograph showed an age-appropriate mature radius adjacent to the ulna in the left upper limb, indicating that the duplication did not involve the forearm bones (Figure 1B). Normal morphology was observed postaxially in the fingers’ phalangeal and metacarpal regions. A prominent dysplasia was observed at the preaxial index (I) finger’s metacarpal base. Preaxial middle (M) and ring (R) fingers showed normal development (Figure 1C).

As described above, our case belongs to type III, the duplication group, according to the IFSSH classification. Al-Qattan’s classification placed it in subgroup A of group 3, meaning intermediate type, because in addition to the duplication of the fingers, a developed radius was present alongside the ulna. After primary consultation with the patient’s parents, after the patient reached the age of one, reconstructive surgery was performed to achieve proper functionality and satisfying aesthetic results.

### 2.2. Surgical Method

From the three preaxial triphalangeal fingers, the operation aimed to create a finger representing the thumb to provide the required functionality and aesthetics. During the design phase, the M was considered a replacement for the thumb because of its well-developed metacarpus, flexor, and extensor muscles. However, the I was finally chosen because of its size and position. 

**Figure 2:**
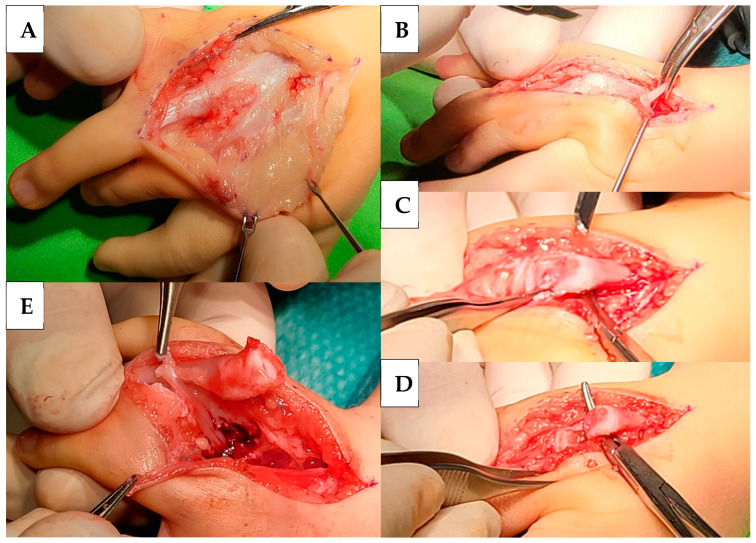
A skin incision and the common extensor tendon that branches at the fingers (**A**). Preaxial I metacarpus mobilisation (**B**). Preaxial M finger metacarpus before (**C**) and after (**D**) resection. The forceps show the point of the resection (**C**) and the elevated and shortened metacarpus (**D**). Flexor and extensor tendons of the preaxial I finger were attached to the flexor and extensor tendons of the remaining finger (**E**).

Extensor apparatuses were explored through a skin incision parallel to the longitudinal axis of M. Through that incision, it was observed that the three preaxial fingers share a common extensor tendon, which branches out in three directions as it reaches the fingers (Figure 2A). After the detachment of the extensor tendons, the metacarpus of the I was mobilised from the surrounding tissues (Figure 2B), followed by the head resection of the metacarpus of the M (Figure 2C,D). Then, the flexor apparatus was also detached (Figure 2E).

**Figure 3:**
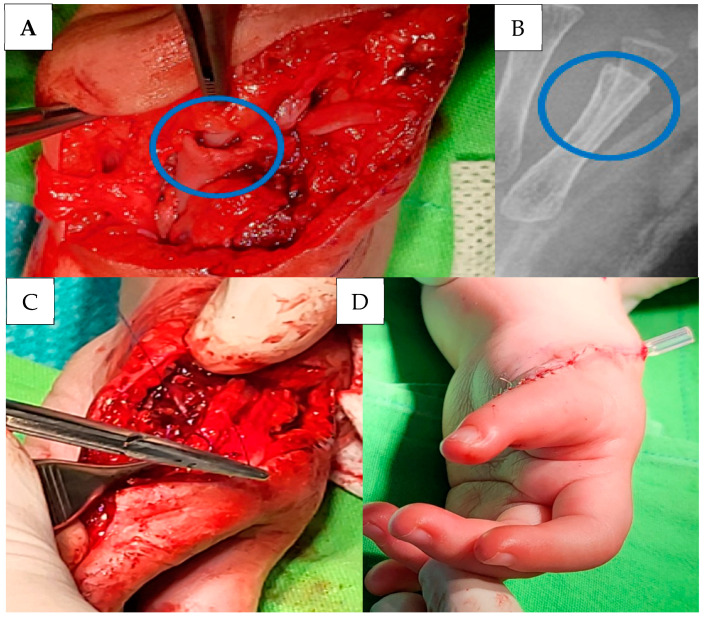
Intraoperative image (**A**) and post surgical radiograph (**B**) showing the metacarpus of the preaxial I wedged into the metacarpus of the preaxial M. Repositioning of the extensor tendon of the preaxial I to the extensor tendon of the remaining finger (**C**). Closure of the surgical wound (**D**).

Following the muscles’ separation, the R was removed together with its metacarpus, and the M was amputated from the proximal phalanges as well. The metacarpus of the I was wedged into the M resected metacarpus to correct the dysplasia. (Figure 3A,B). After that, we rotated the remaining finger to an opposition position. Well-developed extensor and flexor tendons of the M were repositioned to the less developed extensor and flexor tendons of the remaining finger to facilitate the development of its proper range of motion and to fix the position of the wedged bone ends internally by pulling them into each other (Figure 3C). A drain was inserted and then the wound was closed with intracutaneous stitches. The drain was removed two days after the operation (Figure 3D). At the end of the surgery,xx a cast was applied to fix the position of the modified finger externally.

### 2.3. Post Surgical Therapy

In the post surgical period, the patient received antibiotic prophylaxis for 5 days in the form of 3 × 5 mL Augmentin (Amoxicillin + Clavulanic Acid) syrup (125 mg). She had no fever. During post surgical follow-up, we prescribed physiotherapy, supplemented by psychotherapy. The first was necessary for the development of proper physical functionality and ranges of motion, and the latter for the psychological processing of the child’s changing body image. Physiotherapy treatment consisted of playful exercises with the child and parent together in order to motivate the patient to use her modified hand and improve her grip.

## 3. Results

### Post Surgical Follow-Up

**Figure 4:**
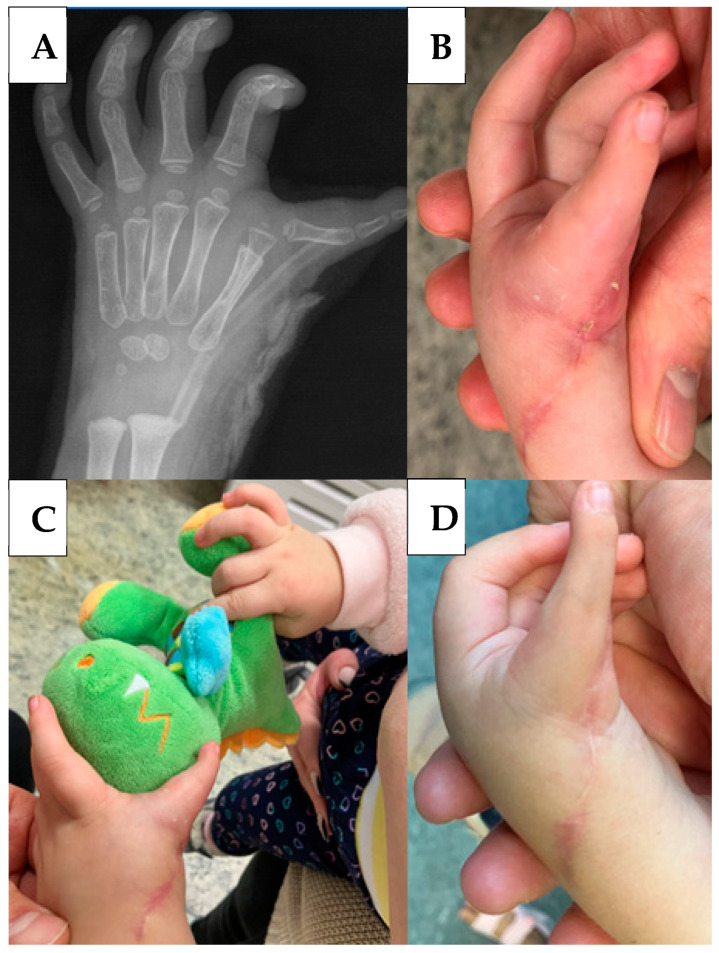
Control X-ray in the fourth week. The metacarpus of the preaxial I wedged into the metacarpus of the preaxial M can be visualised (**A**). The surgical wound (**B**) and the grasping movement (**C**) in the second month. The surgical site sixth months post surgery (**D**).

The first bandage change was performed the day after surgery, where minimal haematoma was visible, although no significant swelling nor vascular or neurological damage was observed. By the tenth day, the surgical wound had completely healed (Figure 4B). A control examination was performed in the first, fourth, and sixth month, which revealed the following findings: In the metacarpophalangeal (MCP) joint of the formed remaining finger, flexion–extension, opposition, and abduction was adequate, and the child had age-appropriate use of the modified left hand, good catching movement, and the same gripping force as the opposite hand (Figure 4C,D).

## 4. Discussion

Ulnar dimelia is a disorder affecting the development of the upper limb. Less than 70 cases of the disease have been reported worldwide. The cases presented below were found to be closest to our own case during a search of the literature. Common to all three cases is that the authors highlight the importance of personalised surgical therapy. This is based on the highly variable anatomical presentation of the pathology, which is described by the Al-Qattan classification.

A. Afshar and colleagues presented the case of a four-year-old child with unilateral ulnar dimelia without axis symmetry. Two ulnae were observed in the right forearm of the child with the absence of a radius and a radially deviated wrist. Four developed postaxial and two preaxial triphalangeal fingers were present on the hand, with a complete absence of the thumb. In the elbow joint, flexion–extension was limited and pronation–supination was not actively possible. Opposition was not possible due to the absence of the thumb. Surgical management was performed to achieve adequate functionality and aesthetic outcomes. During the operation, the first preaxial finger was amputated, then its lateral and medial interosseous muscles and the lateral interosseous muscle of the second preaxial finger were detached from their insertion point and attached to the remaining finger so that these muscles could perform an abductor function. The point of attachment of the second finger’s medial interosseus had been changed to allow for an adductor function of the remaining finger. The amputated preaxial finger’s superficial flexor was utilised to reinforce the abductor function, while the deep flexor tendon supported the extensor function [5].

González-Pola and his team presented the case of bilateral ulnar dimelia. In the patient’s forearms, a well-developed ulna and radius were observed. In both hands, three postaxial fingers with normal development and another three triphalangeal fingers with ulnar deviation were present. Additionally, elbow and wrist joint movements were adequate, but opposition was not possible in this case either due to the absence of the thumb. In order to achieve adequate functionality, they also planned surgical therapy, which was performed in two sessions six months apart. During the operation, the second preaxial finger was removed from the carpometacarpal joint following the separation of the muscle flaps. Then, the first preaxial finger was rotated to an opposing position and was fixed in this position with absorbable sutures, and the insertion point of the common extensor tendon was repositioned to perform an abductor function in the movement of the remaining finger [15].

We present the case of a one-year-old girl born with unilateral ulnar dimelia, classified as group 3/A according to the Al-Qattan classification. Two concepts were considered during the design phase of the surgery. Firstly, the M was considered suitable for the replacement of the thumb due to its developed metacarpus, extensor, and flexor apparatus. However, in its opposition position, the finger proved to be too long, which would not have allowed for a proper gripping pattern. In addition, the complete removal of the I would have caused further difficulties due to the abnormally positioned neurovascular structures. The second idea was to keep the I intact while amputating the M. In this case, the metacarpal and the muscular underdevelopment would have reduced the amount of gripping force. Eventually, a combination of these two ideas resulted in the final surgical solution, in which the I was retained, but the metacarpal wedge corrected the metacarpus underdevelopment. The transposition of the interosseous muscle presented by A. Afshar was not necessary because the repositioned tendon of the M provided adequate gripping force and abduction for the remaining finger. The surgery was performed in a single session, unlike the case presented by González-Pola and his team, in order to avoid the child undergoing the risk of a second surgery and its anaesthetic complications. The examples also demonstrate that due to the diverse anatomical background of the disease, a personalised surgical therapy should always be chosen with the main aim of achieving the proper functionality and the required aesthetics.

## 5. Conclusions

Ulnar dimelia is a rare congenital malformation with a very diverse appearance. Its treatment is primarily surgical, and the most crucial factor is to achieve the correct functionality followed by an aesthetically pleasing result that is satisfactory for the child and their parents. To attain our aim, we need to meticulously tailor individualised operative solutions for each child. The appropriate time for surgery is above the age of one year when the anatomical structures are already large enough to allow for surgery. However, it must be performed under the age of two to take the most advantage of the brain plasticity that comes with youth. Finally, it is essential to use physiotherapy in combination with psychotherapy, because the most important thing is that the child does not negate the modified hand and uses it as it was intended.

## Figures and Tables

**Table 1:** Classification of congenital hand deformities (IFSSH).

Type	Description
I.	Failure of formation
II.	Failure of differentiation
III.	Duplication
IV.	Overgrowth
V.	Undergrowth
VI.	Constriction band syndrome
VII.	Generalised anomalies and syndromes

**Table 2:** Classification of mirror hand—multiple hand spectrum (Al-Qattan MM et al.) [1].

Type	Name	Clinical Features
1	Ulnar dimelia	Multiple fingers with two ulnae: Type A: each ulna is well-formed; Type B: the preaxial ulna lacks the styloid process or is hypoplastic.
2	Intermediate type	Multiple fingers with two ulnae (one of the ulna is vestigial) and a radius.
3	Intermediate type	Multiple fingers with one ulna and a radius: Type A: the radius well-formed; Type B: a hypoplastic radius.
4	Mirror hand syndrome	Bilateral multiple fingers in complex syndactyly: Type A: Laurin–Sandrow syndrome = the forearm contains two ulnae; Type B: Martin syndrome = the forearm contains an ulna and a radius.
5	Multiple hand	Complete duplication of the hand including the thumb with a normal forearm.

## Data Availability

All related data are presented or referenced in the article.
